# MicroRNAs as Potential Regulators of Immune Response Networks in Asthma and Chronic Obstructive Pulmonary Disease

**DOI:** 10.3389/fimmu.2020.608666

**Published:** 2021-01-08

**Authors:** José A. Cañas, José M. Rodrigo-Muñoz, Beatriz Sastre, Marta Gil-Martinez, Natalia Redondo, Victoria del Pozo

**Affiliations:** ^1^ Immunoallergy Laboratory, Immunology Department, Instituto de Investigación Sanitaria Fundación Jiménez Díaz (IIS-FJD), Madrid, Spain; ^2^ CIBER de Enfermedades Respiratorias (CIBERES), Madrid, Spain

**Keywords:** chronic respiratory diseases, systems biology, microRNAs, asthma, chronic pulmonary obstructive disease

## Abstract

Chronic respiratory diseases (CRDs) are an important factor of morbidity and mortality, accounting for approximately 6% of total deaths worldwide. The main CRDs are asthma and chronic obstructive pulmonary disease (COPD). These complex diseases have different triggers including allergens, pollutants, tobacco smoke, and other risk factors. It is important to highlight that although CRDs are incurable, various forms of treatment improve shortness of breath and quality of life. The search for tools that can ensure accurate diagnosis and treatment is crucial. MicroRNAs (miRNAs) are small non-coding RNAs and have been described as promising diagnostic and therapeutic biomarkers for CRDs. They are implicated in multiple processes of asthma and COPD, regulating pathways associated with inflammation, thereby showing that miRNAs are critical regulators of the immune response. Indeed, miRNAs have been found to be deregulated in several biofluids (sputum, bronchoalveolar lavage, and serum) and in both structural lung and immune cells of patients in comparison to healthy subjects, showing their potential role as biomarkers. Also, miRNAs play a part in the development or termination of histopathological changes and comorbidities, revealing the complexity of miRNA regulation and opening up new treatment possibilities. Finally, miRNAs have been proposed as prognostic tools in response to both conventional and biologic treatments for asthma or COPD, and miRNA-based treatment has emerged as a potential approach for clinical intervention in these respiratory diseases; however, this field is still in development. The present review applies a systems biology approach to the understanding of miRNA regulatory networks in asthma and COPD, summarizing their roles in pathophysiology, diagnosis, and treatment.

## Introduction

**Figure 1:**
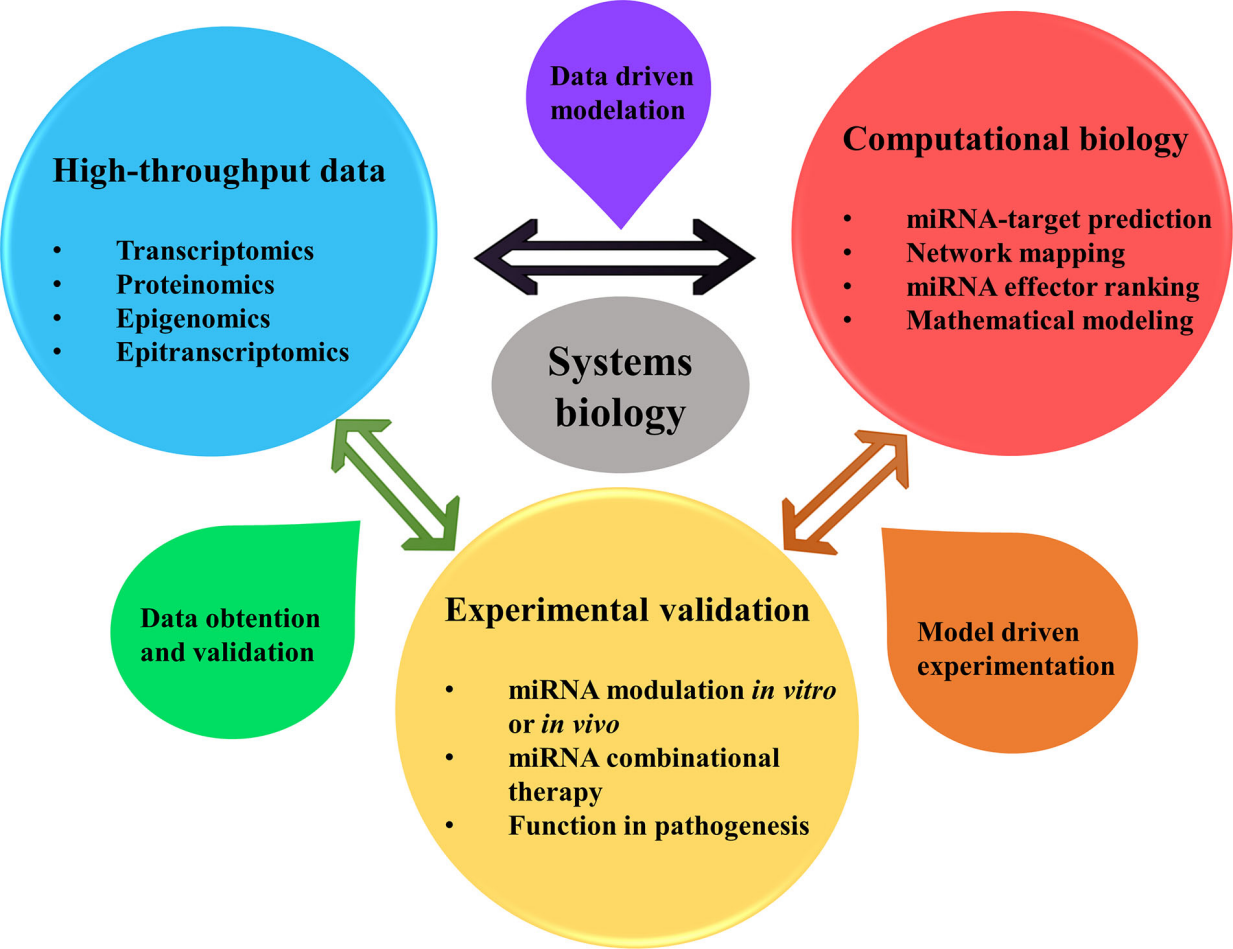
Systems biology approaches allow for better understanding the roles of miRNAs in pathophysiology, diagnosis, and treatment of asthma and COPD. Being miRNAs regulators of multiple genes expression affecting thus several pathways simultaneously, a comprehension of the global picture of their regulation is in need. System biology approach helps in this matter, by using a data driven method for creating computational models from previous data of high-throughput experimentation (transcriptomics, proteomics, epigenomics and epitranscriptomics) that could be obtained from the literature. Network mapping modeling allows determining the interaction between miRNAs, genes (or proteins) and phenotype and clinical data. Besides, mathematical models can help finding key miRNAs with power to alter the core of cellular pathway regulation and performance. After this, model driven experimentation allows to confirm the predicted targeting by miRNAs in cooperation by their modulation *in vivo* or *in vitro*, uncovering their role in the disease and their use in therapy. Finally, this new data obtained and validated can be summed up to confirm previous high-throughput results, enlarging the available data of miRNA regulation in asthma or COPD.

Chronic respiratory diseases (CRDs) like asthma and chronic obstructive pulmonary disease (COPD) are complex and heterogeneous diseases that pose a challenge for investigation and management. Multiple environmental factors and genetic predisposition modulate asthma and COPD phenotypes and severity, as well as how a patient responds to treatment. In these diseases, genomics, metabolomics, epigenomics, and transcriptomics engage in intricate interaction at the cellular level. Systems biology tries to construct models or approaches throughout the network that reveal the underlying biology and help to understand living systems. Traditionally, gene modulation and cell-signaling networks have been thought to be the main regulatory systems in cells, and RNAs have been considered molecules that only codify genetic information to protein synthesis. However, this idea is changing due to the recent advances in non-coding RNAs, such as microRNAs (miRNAs) (1). MiRNAs are 18–22 nucleotides in length and block protein translation by RNA-miRNA interaction (2). These small RNAs regulate hundreds to thousands of genes, offering a broad combinatorial possibility and constituting complicated regulatory networks. As a result, systems biology approaches are essential to understand miRNAs functions in complex diseases such as asthma and COPD, combining data from high-throughput experiments with computational models for performance of data driven modeling and model driven experimental methods (**Figure 1**).

New procedures have been applied in this topic, particularly to determine the coordinate function of miRNAs in cancer. Lai et al. used a systems biology approach to unravel the role of miRNAs therapeutics in this disease (3, 4). This approach highlights the importance of high-throughput experiments to determine from the same biological experiments the transcriptome of miRNAs and their gene targets, with further exploration by proteomic data, or immunoprecipitation-based analysis, as this is very helpful in validating the huge amount of predicted miRNA-gene interactions detected *in silico* by diverse bioinformatic tools reviewed by Gomes et al. (5). After getting this data, then it is easier to apply computational biology approaches, most of them based on previously validated data for gene-miRNA interaction determination. After this, system biology comes in, because as previously stated it helps in creating maps of miRNA-gene-pathway interactions that may have an actual function in cell physiology (6). The actual mapping can be further detailed by introducing the regulation that occurs on the controllers (miRNAs) themselves, as we have to take into account miRNA biogenesis and structure, epigenetics, epitranscriptomics, transcription factor circuits and super enhancers, all of them modulating miRNAs functions in diseases (7).

In this review, we will focus on asthma and COPD, two of the most common CRDs worldwide, providing an overview about those molecular pathology mechanisms by miRNAs. Additionally, we will explore new insights in the field of miRNAs as biomarkers of these diseases. Lastly, we will highlight altered after specific treatment for each disease and discuss clinical advances in the use of miRNAs. With this review we want to set the foundations of actual data of miRNAs as regulators and biomarkers of chronic respiratory diseases, being able to serve as a guide for future application of complex system biology approaches to determine the actual combined effects of this miRNAs in these diseases, seeing the big picture of the pathophysiology.

## Pathophysiology of Asthma and COPD

CRDs are an important cause of morbidity and mortality worldwide. According to the World Health Organization, the most common CRDs are asthma, COPD, lung hypertension, and occupational lung diseases (8). It is estimated that more than 300 million people worldwide have asthma and 3 million people die each year from COPD, accounting for an estimated 6% of total deaths globally (8).

The causes triggering the development of CRDs are diverse. Asthma is a multifactorial and heterogeneous disease, and a variety of risk factors have been linked to this disease such as genetics, atopy, and recurrent viral infections (9). Additionally, tobacco has been described as the main cause of COPD, though exposure to other toxic substances such as air pollution originated from biomass fuel has been also linked to COPD (10, 11). Independently of origin, CRDs are characterized by the inflammation and obstruction of the lower respiratory tract due to a hyperresponse of the immune system accompanied by cellular infiltration (12, 13). In allergic asthma, the predominant leucocytes are eosinophils, with a triggered type 2 immune response with high abundance of interleukin (IL)-4, IL-5, and IL-13 (14, 15); however, in COPD the most abundant cellular populations are neutrophils, macrophages, and T lymphocytes (11, 13).

Asthma presents with high variability among patients, thus posing a challenge for the improvement of diagnostic and therapeutic tools (16). Asthma is characterized by chronic airway inflammation, mucus hypersecretion, and bronchial hyperresponsiveness and the presence of respiratory symptoms such as wheezing, shortness of breath, chest tightness, and cough (17). Airway inflammation and structural remodeling together with reversible airflow obstruction and airway hyperreactivity are the main distinctive findings of asthmatic disease (18). In addition, asthma encompasses several disease variants, meaning that it can be stratified into several phenotypes and endotypes. Phenotyping and endotyping of asthma with the use of induced sputum or peripheral blood cytology can facilitate responsiveness to treatment, specify the pathogenic mechanisms, and anticipate risks. These features attest to the complexity of asthmatic disease and the numerous factors involved in its pathophysiology, suggesting that systems biology can aid in understanding the key factors implicated in molecular networks.

COPD is a multifactorial and heterogeneous disease that affects millions of people worldwide (19). This pathology is a major cause of chronic morbidity and mortality, and many people bear the burden of this disease for years. COPD encompasses small airway obstruction, emphysema, and chronic bronchitis, and it is characterized by chronic inflammation of the airways and lung parenchyma with progressive and irreversible airflow limitation (20). Symptoms include dyspnea, cough, and sputum production (21). The phenotypic characterization of COPD patients may allow for better risk stratification and personalization of therapy (22). Airway damage in COPD is triggered by dust, fumes, vapors, or gas, but the primary factor is exposure to tobacco smoke (23). Cigarette smoke alters both innate and adaptive immunity by upregulating cytokines (IL-1, IL-6, IL-8, tumor necrosis factor [TNF]-α…) (24, 25), and modifying the physiological function of alveolar macrophages (26), dendritic cells (27), neutrophils (28), and natural killer cells (29). Smoking also modifies the behavior of the epithelium by increasing mucin production (MUC5AC) (30) and disrupting epithelial cell-cell junctions, thus increasing the permeability of the epithelial barrier (31).

It has been shown that besides altering the normal physiology of the airways, cigarette smoke may change the epigenetic landscape, and these changes can be passed on to future generations through inheritance (32). Several studies show that COPD causes certain epigenetic changes in the lungs (33, 34), and many of these changes are likely due to tobacco smoke exposure (35), such as *F2RL3* methylation, which is associated with smoking behavior and high mortality (7, 36). These epigenetic changes can be targeted as a possible therapy; current genetic editors like zinc-finger nucleases and CRISPR-Cas9 can be coupled to them and enzymes to rewrite epigenetic markers induced by tobacco smoke or related to COPD pathophysiology (37, 38).

## MiRNAs in Lung Diseases

**Figure 2:**
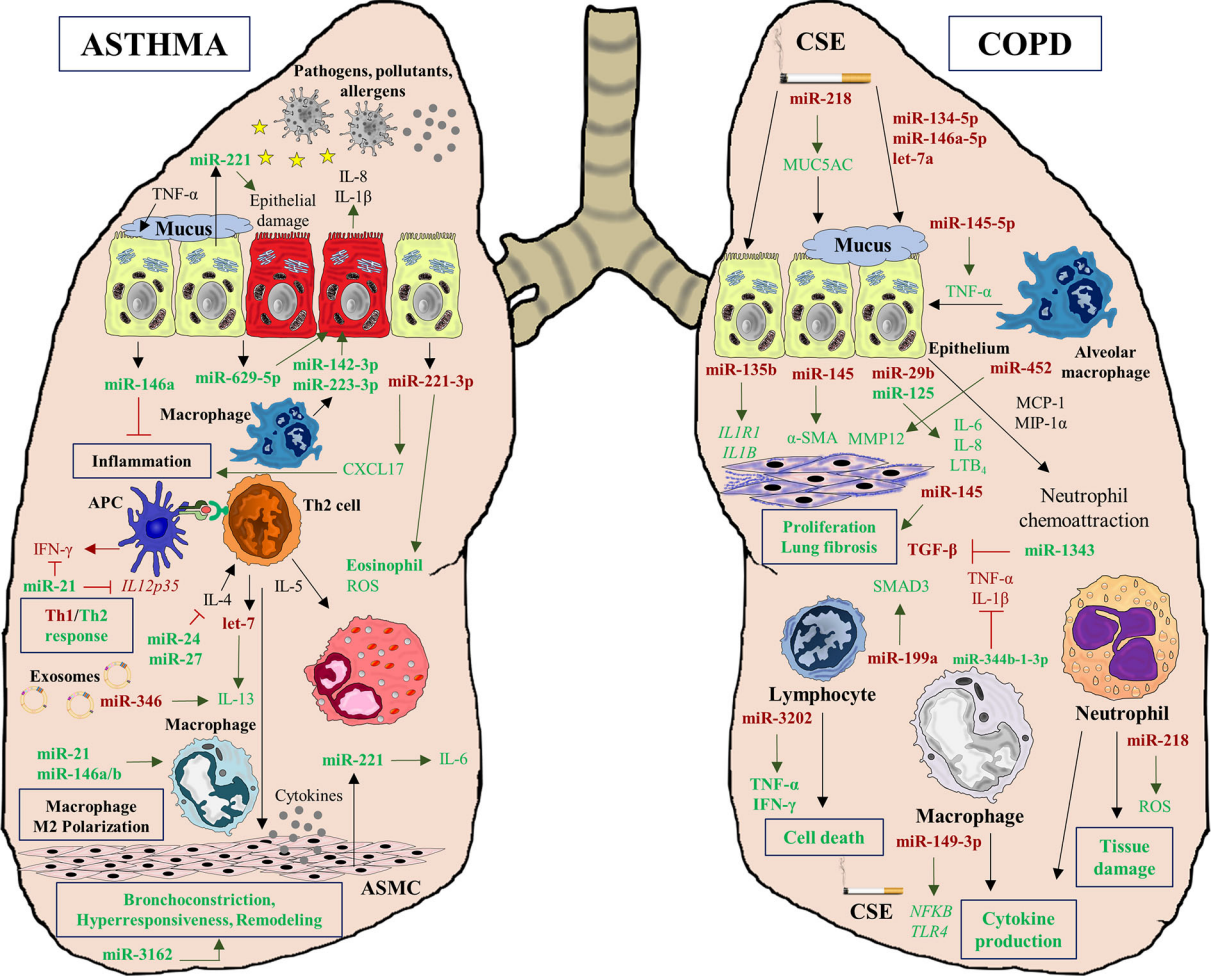
MiRNA interactions in asthma and COPD diseases. MiRNAs play a crucial role, regulating multiples processes characteristics of both pathologies. In asthma, deregulation of multiples miRNAs affects to inflammatory processes (miR-221-3p), Th1/Th2 response imbalance (miR-21), cytokine production (miR-629-5p, miR-142-3p, and miR-223-3p), epithelial injury (miR-221), macrophage polarization to M2 phenotype (miR-146a/b, miR-21) and airway remodeling (miR-3162). However, miRNAs also can alleviate inflammation (miR-146a) and cytokine decrease production (miR-24 and miR-27). In COPD pathogenesis, miRNAs can be altered by CSE exposition and they are involved in mucus hypersecretion (miR-218) and cytokine production (miR-149-3p). Moreover, several deregulated miRNAs are implicated in lung fibrosis (miR-135b, miR-145, and miR-452), and in tissue damage (miR-218). Similarly to asthma, in COPD some miRNAs can act as regulators of inflammation decreasing the secretion of several cytokines such as TGF-β (miR-1343), TNF-α, and IL-1 β (miR-344b-1-3p).

The complex interplay between genetics, epigenetics, protein synthesis, and immune response in asthma and COPD is actually even more intricate when another layer of regulation is introduced: miRNAs. These small molecules or noncoding RNAs are capable of regulating the gene-protein expression of immune system performance (39) and structural airway homeostasis and function (40). Moreover, miRNAs can regulate epigenetic modulators and be regulated by epigenetic changes as well (41). MiRNAs are therefore essential players in the physiopathology of both diseases, creating complex networks and interactions among diverse factors (genes, proteins, cells) that play a role in these pathologies (**Figure 2**).

It is important to differentiate between two kinds of miRNAs: intracellular and extracellular (found inside extracellular vesicles such as exosomes, microvesicles, and apoptotic bodies) (42). Intracellular miRNAs regulate several cellular pathways, and their expression is tissue- and disease-specific, so they have been widely used as prognostic and diagnostic biomarkers of different pathologies, including viral infections, cancer, cardiovascular and allergic diseases (43, 44). Also, circulating miRNAs have been studied and used as biomarkers due to their molecular properties (resistance to degradation and ubiquity) (45).

### MiRNAs as Asthma Biomarkers

Circulating cell-free miRNAs can be found in serum or plasma incorporated into extracellular vesicles, such as exosomes, and in ribonucleoprotein complexes. There are many studies showing miRNA deregulation in asthmatic patients (**Table 1**). It is known that several miRNAs are increased in serum samples including miR-21, miR-145, miR-146a, and miR-338, among others (45, 70). Likewise, other authors have described downregulation of other serum miRNAs, such as miR-18a, miR-126, and miR-155 (71). However, due to the complex relation between miRNAs and genes (a single miRNA can regulate hundreds of genes), not all miRNAs qualify for use as biomarkers. One solution to this problem may be to use combinations of several miRNAs or a specific miRNA profile to achieve good sensitivity, specificity, and positive and negative predictive values.

**Table 1:** List of miRNAs involved in asthma pathogenesis.

miRNA	Implication	Origin	Expression	Target Gene/Pathway	References
miR-140-3p	CD38 expression, chemokine regulation, inflammation and ASMC proliferation in asthma	ASMC	Downregulated	*CD38, CCL11, CXCL12, CXCL10, CCL5, CXCL8*	(46, 47)
miR-145	ASMC proliferation and migration		Upregulated	*KLF4*	(48)
miR-146a-5p	Mucus production		Downregulated	*UBD, CXCL10, CXCL8, CCL20, UCA1*	(49)
miR-638	ASMC proliferation and migration		Upregulated	*NR4A3, CCND1*	(50)
miR-708	CD38 expression, chemokine regulation, inflammation and ASMC proliferation in asthma		Downregulated	*CD38, CCL11, CXCL10, CCL2, CXCL8*, JNK, MAPK, PTEN/AKT signaling pathways	(47, 51)
miR-146a/b	Regulation of inflammation, macrophage M2 polarization	Epithelial cells, macrophages	Upregulated	*PTGS2, IL1B, NOTCH5*	(52–56)
let-7 family	Asthma biomarker	BALF-derived exosomes	Downregulated		(57)
miR-126	Asthma progression		Upregulated	*DNMT1*	(58)
miR-200 family	Asthma biomarker		Downregulated		(57)
miR-346	Airway inflammation, T helper cell differentiation		Downregulated	*IL13*	(59)
miR-574-5p			Downregulated	*IL5RA*	(59)
let-7	Regulation of asthmatic hyperresponse	Lung	Upregulated	*IL13*	(60)
miR-24	Cytokine regulation		Upregulated	IL-4 production pathway	(61)
miR-27			Upregulated	*GATA3*	(61)
miR-16	Asthma biomarker	Plasma	Upregulated		(45)
miR-125b			Upregulated		(45)
miR-133b			Downregulated		(45)
miR-206			Upregulated		(45)
miR-144-5p	Asthma biomarker	Serum	Upregulated		(43)
miR-155			Upregulated		(62)
miR-185-5p			Upregulated		(43)
miR-320a			Upregulated		(43)
miR-1246			Upregulated		(43)
miR-485-5p	Pediatric asthma		Upregulated	*SPRED2*	(63)
miR-3162-3p			Upregulated	*CTNNB1*	(64)
miR-221	Pediatric asthma, Epithelial cell injury	Serum, epithelial cells	Upregulated	*SPRED, SIRT1*	(63, 65)
miR-21	Imbalance Th1/Th2 response, macrophage M2 polarization	Serum, lung, macrophage	Upregulated	*IL12p3, IRF5, CSF1R*	(43, 52, 54, 62, 66, 67)
miR-142-3p	Neutrophilic asthma	Sputum	Upregulated	MAPK, NOD-like receptor, Toll-like receptor, JAK-STAT, and the TGF-β signaling pathways	(68)
miR-223-3p			Upregulated		(68)
miR-629-3p			Upregulated		(68)
miR-221-3p	Regulation of eosinophil counts and ROS production		Downregulated	*CXCL17*	(69)

In 2016, Panganiban et al. established a differential miRNA profile among asthmatic patients, non-asthmatic patients with allergic rhinitis, and non-asthmatic non-allergic subjects (45). In their study, the researchers found 30 miRNAs in plasma that were differentially expressed among three groups, showing six miRNAs (miR-125b, miR-16, miR-299-5p, miR-126, miR-206, and miR-133b) with a high predictive value when differentiating allergic and asthmatic status. Moreover, some of these circulating miRNAs grouped asthmatic patients into two clusters according to the number of peripheral blood eosinophils. Finally, they demonstrated that circulating miRNAs could be used to diagnose both allergic rhinitis and asthmatic patients and characterize asthma subtypes. Milger et al., in 2017, identified some possible plasma miRNA candidates as biomarkers in a murine model of asthma (72). These miRNAs were validated in a different cohort of healthy subjects and asthmatic patients, using a regularized logistic regression model to identify five miRNA ratios that are able to differentiate allergic asthmatics from controls with an area under the curve (AUC) of 0.92. However, this miRNA signature did not differentiate asthma sub-phenotypes.

Our group recently described an eosinophil miRNA profile for asthma diagnosis based on statistical models (43). First, we described a miRNA signature from peripheral blood eosinophils, which is able to differentiate asthmatic subjects from healthy controls. Among deregulated miRNAs, we found upregulation of miR-21 and miR-146a/b, which have been associated with asthma and allergic diseases (73). In addition, this molecular profile grouped the asthmatic population into two principal clusters, which are distinguishable by the number of peripheral blood eosinophils and serum periostin levels. Then, we evaluated these miRNAs in serum and found that miR-1246, miR-144-5p, miR-320a, miR-185-5p, and miR-21-5p were upregulated in asthmatic patients. Specifically, miR-185-5p was related to asthma severity. Combination of miR-185-5p, miR-144-5p, and miR-1246 in a logistic regression model distinguished healthy individuals from asthmatics with an AUC of 0.86, and a specificity and sensitivity of 0.89 and 0.77, respectively. Serum expression of miR-320a, miR-185-5p, and miR-144-5p was used to classify subjects into different asthma disease phenotypes (intermittent, mild persistent, moderate persistent, and severe persistent) in a random forest model, showing that miRNAs can be used for asthma diagnosis and severity classification. Unfortunately, to date no more studies about specific miRNAs of eosinophils have been conducted, making this is a field of potential research focus.

Elkashef et al. have reported the use of miR-21 and miR-155 as biomarkers in bronchial asthma (62). They showed that both miRNAs are higher in serum of individuals with eosinophilic asthma than healthy subjects. Through a receiver operating characteristic curve with both miRNAs, the authors obtained high values for sensitivity, specificity, and AUC. Thus, they proposed that these miRNAs could be used as non-invasive biomarkers in asthma diagnosis and response to therapy.

As mentioned previously, another important factor in asthma pathogenesis is the role that exosomes exert in the disease (74). These particles are nanovesicles, with a diameter of 30–150 nm, and their main role is linked to intercellular communication. It is worth highlighting that these small vesicles contain miRNAs in addition to DNA, proteins, and lipid mediators. Thus, some circulating miRNAs have been associated with exosomes and it has been observed that these exosomal miRNAs play a critical role in asthma pathogenesis (75, 76).

It is important to take into account that exosomes can be located in multiple biological fluids including serum, sputum, bronchoalveolar lavage fluid (BALF), urine, breast milk, etc., and that exosomes are associated with multiple pathological processes, including asthma (16). In 2013, Levänen et al. found significant differences in a set of exosomal miRNAs from BALF between mild asymptomatic asthmatic patients and healthy subjects, including miRNAs of the let-7 and miR-200 families (57). Later, Gon et al. showed 139 exosomal miRNAs from BALF deregulated in a house-dust mite murine model compared to control mice (59). Also, they observed that 54 altered miRNAs were common between exosomes and lung tissues. Using computational analysis, the authors then found that 31 genes were the targets of these miRNAs, including important genes in asthma pathogenesis such as *IL13* and *IL5RA*.

Different studies have been conducted in exosomes from serum (58, 77). These showed that several miRNAs, including miR-125b and miR-126, were altered in these nanovesicles between asthma condition and normal status. According to the authors, these results can be applied to the use of novel diagnostic strategies.

#### Sputum miRNAs Characterize the Inflammatory Focus

While it is clear that serum/plasma miRNAs are good non-invasive biomarkers, miRNAs from sputum samples could also be used as diagnostic tools. However, the number of studies in this field reduced drastically and only a few research articles address this topic (**Table 1**). One of them was performed in 2018, conducted by Zhang et al. (69). In this study, the authors found that sputum and plasma miR-221-3p levels were significantly decreased in asthmatics compared with healthy subjects. Furthermore, there was a positive correlation between plasma and induced sputum miR-221-3p levels and values of this miRNA in epithelial cells, just like a negative correlation with the eosinophil percentage in sputum, the number of eosinophils in bronchial biopsies, and fraction of exhaled nitric oxide levels. The study highlighted that sputum miR-221-3p levels were increased in asthmatic patients after 4 weeks of inhaled corticosteroid (ICS) treatment, compared with asthmatic patients at baseline. With these data, the researchers proposed that sputum miR-221-3p could be used as a biomarker for airway eosinophilic inflammation and response to treatment. In another study performed in 2016, Maes et al. showed a different miRNAs profile in sputum samples among healthy subjects, patients with mild-to-moderate asthma, and patients with severe asthma (68). They presented three miRNAs that were increased in sputum samples from patients with severe asthma, which are related to neutrophilic asthma phenotype (**Table 1**).

### MiRNAs for Characterization of COPD

Study of miRNAs has been rapidly extended to respiratory diseases including COPD, likely due to research showing that cigarette smoke acts as a modulator of miRNA regulation in samples such as lung tissue (78, 79), serum (80) and sputum (81) (**Table 2**). Therefore, similarly to asthma, there are miRNAs that can serve as biomarkers for COPD against healthy conditions in different biofluids. MiRNAs also regulate the expression of genes in COPD. Several miRNAs have been implicated in the physiopathology of COPD (89, 149). However, it is also important to mention that some miRNAs are also related to protective functions in COPD pathology when induced by treatment (144).

**Table 2:** List of miRNAs involved in COPD pathogenesis and diagnosis.

miRNA	Implication	Origin	Expression	Target Gene/Pathway	References
miR-452	Control of airway inflammation and remodeling	Alveolar macrophages	Downregulated	*MMP12*	(82)
miR-344b-1-3p	Modulator of the immune responses by CSE		Upregulated	*TLR2*	(83)
miR-637	Development of pulmonary hypertension	Artery smooth muscle cells	Downregulated	*CDK6*	(84)
miR-197	Vascular remodeling and contraction		Downregulated	*E2F1*	(85)
mir-106a-363 and -106b cluster	COPD diagnosis and severity	BALF, plasma, leukocytes	Upregulated/downregulated	*AKT1, PTEN, MYD88, IRAK4, IL6, TGF-βR*	(86–88)
miR-146a	Inflammation and mucus secretion	BECs, fibroblasts, macrophages, plasma, lung tissue	Upregulated/downregulated	*IRAK1/TRAF6, PGE2, COX2, PDE7A, IL-1R kinase-1*	(52, 89–95)
miR-195	Cytokine production and inflammation	BECs, lung tissue	Upregulated	*PHLPP2*	(96)
miR-132	Direct correlation to FEV1/FVC%	BECs, monocytes, serum	Upregulated	*SOCS5*	(97)
Profile of 9 miRNAs	Lung cancer prediction in COPD patients	Blood cells	Downregulated	*SRCAP, DCTN5, ULK1, and SEMA7A*	(98)
miR-183 (-5p)	Increases disease severity and pathogenesis	Blood, lungs, smooth muscle cells, leukocytes	Upregulated/downregulated	*BKCaβ1, Autophagy, TLR, NSCLC, cardiomyopathy*	(88, 99)
Profile of 5 miRNAs	Differentiating healthy, asthma and COPD	Breath exhaled condensate	Downregulated in asthma	*IL-13, IL-5, GATA3, FcϵR1 β, IL-1 β, MMP-1, Mucin-1*	(100)
miR-29b	Regulation of cytokine expression	Bronchial epithelial cells	Downregulated	*BRD4*	(101)
miR-10a-5p	Pathobiology of COPD and asthma		Upregulated	*FOXO3 and PDE7A*	(94)
miR-132-212, -17-92, -192-194 clusters	COPD diagnosis against lung cancer	BALF	Downregulated	*AKT1, ERBB2, KRAS, PTEN, MYD88*	(87)
miR-191	Endothelial injury and inflammation	Circulating endothelial microparticles released by CSE	Upregulated		(102)
miR-638	Regulation of oxidative stress response	Emphysematous lung tissue	Upregulated	*ADAM15, HDAC5, APBB1*	(103)
miR-126	Endothelial injury and inflammation	Endothelial microparticles, ECs, UVECs	Upregulated/downregulated	*ATM protein kinase*	(102, 104)
miR-125a (-5p), 125b	Inflammation, severity and AECOPD	Endothelial microparticles, leukocytes, sputum, plasma	Upregulated	*Autophagy, TLR, NSCLC, cardiomyopathy*	(81, 88, 102, 105)
miR-503	Correlate with pulmonary function (FEV1)	Lung fibroblasts	Downregulated	*VEGF*	(106)
miR-335-5p	Modification of fibroblast behavior and function		Downregulated	*Rb1, CARF, and SGK3*	(107)
miR-31-5p	Regulator of mucus hypersecretion	Lung tissue	Upregulated	*ST3GAL2, PITPNM2, ARHGEF15*	(108)
miR-1274a, -424	Emphysema and fibrosis.	Lung tissue and bronchial epithelial cells	Upregulated	*IL-6, TEP1, CAT, TGFβ, and WNT pathway*	(79)
miR-134-5p	Chronic mucus hypersecretion homeostasis		Downregulated	*KRAS*	(90)
miR-150	Suppression of CSE-induced inflammation		Downregulated	*P53*	(109)
miR-223	Emphysema, fibrosis, immunity	Lung tissue, BECs	Upregulated	*IL-6, TEP1, CAT, TGFβ, WNT, HDAC2*	(79, 110)
let-7 family (a,c,d)	Mucus, inflammation, malignancy	Lung tissue, BECs, BEC, sputum, serum	Upregulated/ downregulated	*EDN1, IL-13, TGF- βR, TLR4, c-myc, TNFR-II*	(78, 81, 90, 100, 102, 111, 112)
miR-21	Lung function, inflammation, CRD differentiation	Lung tissue, BECs, exosomes, serum, plasma, BEC	Upregulated/downregulated	*GF-β/Smad, PTEN, PI3K/HDAC2, Notch1, IL-13R, STAT3, IL-1 β*	(95, 100, 113–119)
miR-101	Mucus homeostasis and inflammation	Lung tissue, macrophages	Upregulated	*MKP-1*	(120)
miR-218 (-5p)	Malignant transformation, lung function	Lung tissue, BECs, serum	Downregulated/ upregulated	*BMI1, TNFR1*	(121–124)
miR-199a-5p	Negative correlation with pulmonary function	Lung tissue, PASMCs, Treg	Upregulated/downregulated	*HIF-1α, SMAD3, TGF-β pathway*	(125–127)
miR-146b	Regulating innate macrophage responses	Macrophages	Upregulated	*STAT1*	(53)
profile of 8 miRNAs including miR-135b	Diagnosing AECOPD, inflammation	PBMCs, BECs	Upregulated	*IL-1R1 and IL-1β*	(128, 129)
miR-1273g-3p, -24-3p, -93-5p	Correlated with pulmonary function (FEV1)	Peripheral mononuclear cells	Downregulated	*IL18, IL1B, TNF, NFKBIA, CCL3, and CCL4*	(130)
Profile of 5 miRNAs	Differentiation of COPD and asthma	Plasma	Different expression between COPD and asthma	*Interferon-gamma inducible*	(131)
Profile of 9 miRNAs	Differentiation of COPD	Plasma and airway epithelial cell EVs	5 upregulated and 4 downregulated	*Inflammation, extracellular matrix and remodeling*	(132)
miR-206	Apoptosis, atrophy, inflammation	pvASMCs, limb tissue, fibroblasts	Upregulated	*Notch3, VEGF, HDAC3, HDAC4, IGF-1, SIRT-1, IRAK1*	(133–135)
miR-20a, -28-3p	COPD diagnosis	Serum	Downregulated		(136)
Profile of 9 miRNAs	Environmental factors in COPD subjects		Differentially expressed		(137)
miR-1	Atrophy	Serum / limb tissue	Downregulated / Upregulated	*pAKT, HDAC3, HDAC4, IGF-1, SIRT-1*	(133, 138)
miR-7	COPD diagnosis, inflammation	Serum, ASMCs	Upregulated	*Epac1*	(136, 139)
miR-100 (-5p)	COPD severity	Serum, leukocytes	Downregulated/ upregulated	*Autophagy, TLR, NSCLC, cardiomyopathy*	(88, 136)
miR-34 (a, b, c, c-5p)	Lung function, apoptosis, emphysema	Serum, lung tissue, pmvECs, BECs, sputum	Downregulated/ upregulated	*HIF-1α, Notch-1R, SIRT-1 and 6, SERPINE1*	(81, 125, 136, 140–142)
miR-181a, c	COPD development, inflammation, ROS	Serum, lung tissue, BECs	Downregulated	*MMP, cell growth, apoptosis, NKs, CCN1*	(118, 143)
miR320a, b,d	CRD differentiation, lung function, inflammation	Serum, PBMCs, BECs	Upregulated	*T‐cellR, FoxO, TGF‐β, MAPK, PI3K‐AKT, IL1B*	(130, 144, 145)
miR-30a-3p	Correlate with pulmonary function (FEV1)	Sputum	Downregulated		(81)
miR-145	Differentiating CRDs, fibrosis and immunity	Sputum, serum, ASMCs, BECs, lung tissue, plasma	Upregulated/downregulated	*SMAD3, KLF4 CFTR, KLF5*	(70, 111, 131, 146–148)
miR-338/(-3p)	Differentiation of asthma, COPD and ACO	Sputum, serum, plasma	Upregulated/ downregulated	*RAB14, IGF2R*	(70, 131)
miR-27a	Associated to muscle weakness and atrophy	Vastus lateralis from limb tissue	Upregulated	*HDAC3, HDAC4, IGF-1 and SIRT-1*	(133)

Related to lung function, some miRNAs have shown correlation with worse pulmonary function values in COPD, some of them being directly correlated with forced expiratory volume in the first second (FEV_1_) values (81, 104, 106, 130); while others, correlate negatively (96, 125); and even, in other cases, present a negative correlation with FEV1/FVC ratio (97, 113, 122) (**Table 2**).

Regarding characteristic processes of COPD, fibrosis is almost the main pathological event that occurs in this disease. MiR-1343 reduce transforming growth factor *(TGF)-β* receptors I and II, *SMAD2* and *SMAD3*, which are fibrotic factors (150), whereas miR-145 may have profibrotic effects, inducing differentiation of lung myofibroblasts, while also negatively regulating cytokine expression of airway smooth muscle cells (ASMCs) (146, 147). Mucus hypersecretion, is in turn regulated by several miRNAs in bronchial biopsies by targeting the mucin-related genes (90) (**Table 2**). Other miRNAs, such as miR-101 and miR-144, are upregulated in COPD lungs activating the ERK pathway (120, 151), while miR-15b is also increased in COPD lungs, and the expression of its target *SMAD7* is decreased (79).

MiRNAs are also important in COPD comorbidities like limb-muscle weakness, as it has been shown that miR-1, miR-206, and miR-27a are upregulated in limb tissues from weak muscle COPD (133). Decreased levels of miR-637 in COPD pulmonary artery smooth muscle cells (PASMCs) are related to pulmonary hypertension (84), while miR-197 is involved in ASMCs proliferation and phenotype (85), and miR-183 expression is augmented in blood from COPD and related to disease severity, targeting *KCNMB1* in lungs and smooth muscle cells (99).

Some miRNAs have been associated with emphysema, another COPD hallmark, like miR-638 (103); or miR-223 (110). On the contrary, others present protective effects on emphysema, like miR-452, appearing downregulated in alveolar macrophages from COPD (82), or miR-34c, that is also downregulated in moderate compared to mild emphysematous tissue (140).

#### Cigarette Smoke Induces Tissue miRNA Changes Associated With Malignancy

As previously said, miRNAs are profoundly implicated in the regulation of pathogenesis of respiratory diseases such as asthma and COPD. To begin with, miRNAs are affected by cigarette smoke exposure. Many researchers have studied the effect of cigarette smoke extract (CSE), both *in vivo* and *in vitro*, showing modulations in miRNA expression that are accompanied by the consequent functional effect.

In COPD, control of the inflammation induced by cigarette smoke is critical and is performed by miRNAs like miR-135b or miR-29b, that regulates *IL1R1*, *IL1β*, and IL-8, respectively (128, 152). Indeed, in lung tissue and blood from COPD individuals, several cytokines such as IL-6 or TNF-α are found downregulated by the action of miR-203 (153). MiR-146a plays an important role in COPD as previously commented. It is upregulated in response to cigarette smoke and fine-tunes the immune response by controlling cyclooxygenase-2 (COX-2) expression in fibroblasts (91). MiR-146a is also upregulated in epithelial cells in response to particulate matter and acts as a negative feedback loop that involves IL-6 and IL-8 (NF-κβ signaling) (92). Nevertheless, the function of this miRNA might be more complex, as another study showed that fibroblasts from COPD stimulated with IL-1β and TNF-α produce less miR-146a, resulting in a high prostaglandin E_2_ (PGE_2_) expression (89). This can be supported by data that show that the minor allele of the rs2910164 polymorphism associates with less miR-146a expression and higher PGE_2_ levels (154). This complexity might be explained by how this miRNA is expressed in a cell-specific manner as it seems to be downregulated in COPD sputum but overexpressed in lung tissue (79, 81).

This is not the only association of miRNA polymorphisms in COPD. Some polymorphisms provide resistance, as in rs3746444 (miR-499) and rs11614913 (miR-196a2), both associated with COPD protection, bronchodilator response and, to a lesser extent, COX-2 expression (miR-146 single nucleotide polymorphisms) (154–157).

Another study showed that miR-218-5p is reduced in lung tissue from COPD and healthy smokers and in mice and 16HBE exposed to CSE, and its expression correlates directly with FEV_1_ values. This miRNA performs a protective role by reducing the inflammation in COPD lungs (122, 123), something that has been corroborated by other studies (101, 122–124). Similarly, miR-181c targets *CCN1* and reduces inflammation, reactive oxygen species (ROS), and neutrophil infiltration (143). MiR-483-5p is able to reduce Beas-2B proliferation and alpha-smooth muscle actin (α-SMA) and fibronectin production in fibroblasts (158) just like let-7c, which is decreased in COPD sputum and lungs from mice and rats exposed to CSE (78, 81, 159).

Other miRNAs exert similar effects, like miR-145-5p, which is able to reduce TNF-α, IL-8, and IL-6 (111). Nevertheless, the role of this miRNA is not clear, existing contradictory data about it (148).

In blood samples from smoking COPD patients, miR-149-3p was downregulated, and monocytes exposed to CSE diminished this miRNA expression and upregulated *TLR4* and *NF-κβ*, which increases inflammation (160). This is also observed for miR-3202, which suppresses the increase of TNF-α and interferon gamma (IFN-γ) induced by CSE in lymphocytes (161), and similarly for miR-150, who has a comparable role (109).

The methylation effect induced by CSE was observed in miRNAs from human bronchial epithelial cells (HBECs), where miR-218 and let-7c inhibition is related to HBE malignancy (112, 121, 162). MiRNAs are regulated by epigenetics, but they also regulate the epigenetic landscape, like miR-217. This miRNA is downregulated by CSE and, at the same time, CSE upregulates the long-noncoding RNA (lncRNA) *MALAT1*, which is a target of miR-217 inducing malignancy (163).

Some miRNAs are upregulated directly by CSE, like miR-21, which is induced by hypoxia-inducible factor 1-alpha (HIF-1α) (114, 164), or others, like miR-664a-3p (targets *FHL1*), which is raised in lung tissue and peripheral blood mononuclear cells (PBMCs) from COPD patients, and in Beas-2B cells exposed to CSE (165). Exosomes are also carriers for pathogenic miRNAs, as CSE-treated HBECs produce exosomes carrying miR-21, inducing myofibroblast differentiation and increasing α-SMA and collagen-I through HIF-1α. Interestingly, downregulation of miR-21 in mice prevented remodeling by CSE (113). This miRNA is also implicated in the autophagy and apoptosis produced by CSE in lung tissues of both mice and 16HBE cells, being associated with worse lung function, proving its potential as therapeutic target in COPD (115, 116).

It is known that CSE upregulates miR-34a expression in human pulmonary microvascular endothelial cells and increases their apoptosis by targeting *NOTCH1* receptor (141), although this relation seems to be the inverse in serum from women with COPD exposed to biomass smoke (117). MiR-34a upregulation is also induced by oxidative stress (related to CSE) *via* phosphatidylinositol 3-kinase (PI3K) signaling in HBECs, which downregulates the antiaging-related deacetylases *SIRT1* and *SIRT6* (166), being this result confirmed in another study (142).

In the same way, CSE upregulates miR-206 in human pulmonary microvascular endothelial cells and in COPD patients, inducing cell apoptosis (134). CSE exposure, in both mice and Beas-2B cells, increased miR-130a levels, inducing a decrease in *WNT1* and therefore causing cell injury, proliferation, and migration by regulation of Wnt/β-catenin signaling (167). Similarly, CSE upregulates miR-195 in Beas-2B cells, increasing phospho-AKT and IL-6 synthesis (96). To the contrary, other miRNAs, such as the anti-malignant miR-200c, are indirectly downregulated by CSE and IL-6 through NF-κβ signaling (168).

CSE exposure releases circulating endothelial microparticles that are miRNA-enriched and, when engulfed by macrophages, can inhibit their efferocytosis activity (102). Moreover, expression of *HIF-1α* is controlled by miR-34a and miR-199a-5p, both overexpressed in COPD lungs, and also by miR-186 in fibroblasts, showing the intricacy of miRNA regulation (125, 169). Confirming this complex interplay of miRNA master regulation, miR-199a-5p is downregulated in regulatory T cells from COPD. This miRNA targets the TGF-β pathway, and its aberrant expression may implicate adaptive immunity dysregulation (126). MiR-199a-5p downregulation is also present in monocytes from COPD patients, where the protein unfolding response is activated and involved in COPD pathology (170), while it also seems to regulate pulmonary artery hypertension through targeting of *SMAD3* (127). This account for the very different functions that miRNAs may perform depending on which tissues or cells are present, and how their expression is influenced by the pathological environments, performing a very specifically fine tuning of gene expressing that occurs in a systemic manner.

Monocytes exposed to CSE also upregulate miR-132 expression inducing an increase in epidermal growth factor receptor, IL-1β, and TNF-α. Similar results were observed in 16HBE cells, and indeed this miRNA is upregulated in serum from COPD and smokers (97). In addition, ASMCs have deregulated miRNA expression due to the CSE effect, as seen by the upregulation of miR-7 and the consecutive reduction of its anti-inflammatory target Epac1 in ASMCs and COPD lungs (139, 171). Likewise, fibroblast behavior and function are altered by miRNAs modulated by CSE, adding another layer to the system regulation of miRNAs function in pathological status (93, 106, 107, 135, 172).

Finally, *in vitro* and *in vivo* CSE models showed that the increased DNA damage response due to CSE is also regulated by miR-126, whose downregulation in COPD causes *ATM* protein kinase activation promoting tissue dysfunction and aging (104).

### Differential Expression of miRNAs in Asthma and COPD and Their Roles as Disease Mediators and Biomarkers

The implications of miRNAs in the human asthmatic response have been widely investigated in both pediatric and adult populations. In 2012, a study by Liu et al. showed differences in the miRNA profile between asthmatic children and healthy controls (63). They performed a miRNA microarray to screen for differential expression of miRNAs in the pediatric population and found upregulation of miR-221 and miR-485-3p in asthmatic children. Also, the authors identified a potential target of both miRNAs, *SPRED2*, a negative regulator of different mechanisms in asthma such as airway inflammation and hyperresponsiveness, by modulating IL-5 signaling pathway. These results were confirmed in a murine model of asthma, showing a significant reduction of Spred-2 in asthmatic mice. In the same context, miR-3162-3p was identified as upregulated in childhood asthma implicated in remodeling through β-catenin (64). Wang et al., in 2015, observed an altered miRNAs signature in peripheral blood from patients with childhood asthma, showing that the levels of plasma miR-let-7c, miR-486, and miR-1260a in children with asthma were significantly higher than in healthy individuals (173).

To ascertain how miRNAs are involved in adult response to asthma, several studies in adulthood population have also been performed. Most research compares the miRNAs profile between asthmatics and controls in a variety of sample types. Other studies on miRNAs in asthma in adult population have been reported, describing a number of miRNAs such as miR-20b, miR-138, miR-143, miR-145, and others and their role in adulthood asthma (174).

Examples of miRNA profiles that allow COPD diagnosis, development or differentiation against healthy conditions have been shown in serum (118, 136). Also, a set of nine miRNAs were found to be differentially expressed in serum between healthy, COPD, and a migrant population with COPD, showing that miRNAs may change with exposure to different environmental factors (137).

Plasma levels of miR-106b are associated with COPD patients versus normal smokers (86), and levels of seven miRNAs are distinctive COPD biomarkers distinguishing from healthy subjects and asthma patients, with miR-145-5p being related to severity and miR-338-3p related to smoking COPD (131). Also, in exhaled breath condensate has been described miRNA profiles that are differential between COPD, healthy patients, and asthmatics (100). Discriminating between COPD and similar diseases has also implemented miRNA studies. Blood cell miRNAs (a profile of 14 miRNAs) and clustered miRNAs from BALF can be used as biomarkers for discrimination of COPD against lung cancer (87, 175). Moreover, in blood, nine miRNAs including members of the miR-320 family, which target mitogen-activated protein kinase (MAPK) pathways, can be used for lung-cancer prediction in COPD subjects (98). Finally, the combined expression of hsa-miR-195 and hsa-miR-143, obtained from databases, is able to identify lung cancer compared to disease-free status, but cannot distinguish COPD from lung cancer (176). From peripheral leucocytes, differential expression of miR-106b-5p, miR-183-5p, miR-125a-5p, and miR-100-5p was found in COPD compared to healthy controls, and miR-106b-5p was directly correlated with disease severity alleviation (88). Several studies have focused their interest on miRNAs from specific immune system cells exploring the implication of these structures in processes linked to asthma or COPD. For example, miR-24 and miR-27 have implications for the type 2 response by regulating IL-4 production by T-cells (61). Other miRNAs such as miR-17 and miR-19 have regulatory functions on T cell proliferation and differentiation to Th1, Th17, and regulatory T cells or by modulating type 2 immune response by inducing PI3K, JAK-STAT, and NF-κβ signaling pathways (177). One of the most widely studied is miR-21. The first study that demonstrated the implication of miR-21 in allergic airway inflammation is by Lu et al. (66). The authors observed an upregulation of miR-21 in transgenic mice with allergic inflammation compared to controls. Through predictive algorithms, they identified potential target genes such as *IL-12p35*. This gene is implicated in type 1 immune response; thus, high levels of miR-21 repress the expression of *IL-12p35*, contributing to type 2 polarization characteristic of asthma and other allergic diseases. In another experimental *in vivo* model of asthma published in 2011, Lu et al. observed the preventive role of miR-21 in the expression of IL-3, IL-5, and IL-12 (178). In their study, the group observed that this miRNA may play a role as regulator in type 1/type 2 immune response balance, repressing cytokines of both types of response. In the same way, other miRNAs have been associated with asthma response in murine models, including miR-1, miR-145, miR-150, and miR-155 (179). Also, the let-7 family comprises the most abundant miRNAs in mouse lungs, playing a potent proinflammatory role in asthma (60). In particular, let-7a is an essential regulator of IL-13, which is a key cytokine that induces airway hyperresponsiveness in the lung tissue of asthmatics. Repression of this cytokine can alleviate allergic asthma symptoms. However, mmu-let-7a is markedly suppressed in Th2 cells, allowing IL-13 expression and stimulating the typical type 2 response of asthma pathology.

T lymphocytes are crucial in asthma pathogenesis, specifically orchestrating type 2 immune response. Naïve T cells turn into Th2 cells, releasing a set of cytokines (IL-4, IL-5, and IL-13), which triggers the characteristic processes of asthma (180). In this case, miRNAs related to T cells play an important role in type 2 immune response and asthma pathology. One of these miRNAs is miR-29b, which is involved in the development of asthma. This miRNA indirectly affects to Th2 response by regulating T-box transcription factors and IFN-γ production in T helper cells (181), so a lower expression of this miRNA in asthmatic lung allows a higher production of IFN-γ in order to recover Th1/Th2 balance in asthmatic lungs (182). According to miR-19a, Simpson et al. in 2014 showed that this miRNA is expressed by T cells and promotes Th2 cytokine production by simultaneously targeting inhibitors of the NF-κB, JAK-STAT, and PI3K pathways (177). Also, they observed that miR-19a had higher expression in human airway-infiltrating T cells in asthma. MiR-19a promotes cytokine production, amplifying inflammatory signaling by inhibiting *PTEN*, the signaling inhibitor SOCS1, and the deubiquitinase A20. Another important miRNA linked to T cells is miR-34a. This miRNA has been found to be upregulated in lungs of ovalbumin-induced asthmatic mice (183), modulating *FOXP3*, a master regulator of regulatory T cells.

Macrophages are immune cells involved in a wide range of functions related to innate and adaptive response, including maintenance of tissues and homeostasis. An imbalance between macrophages M1 (classically activated) and M2 (alternatively activated) phenotypes exists, and M2 polarization has been associated with development of asthma (184). Macrophages play a dual role in this disease, contributing to the induction and progression of eosinophilic lung inflammation and airway remodeling, and protecting against both development of neutrophilic inflammation and more severe airway hyperresponsiveness (185). This phenotype is induced by Th2 cytokines (IL-4 and IL-13), upregulating several genes. The role played by several miRNAs in macrophage polarization and their influence in asthma have been established (186). Several studies have demonstrated that miR-146a, miR-146b, and miR-21 promote macrophage polarization toward the M2 phenotype or suppress M1 polarization (52, 53). According to previous research, these miRNAs are upregulated in asthma (54). They act by joining target genes (*NOTCH1*, *IRF5*, and *CSF1R*), inhibiting the inflammatory response (67, 187, 188). In macrophages from COPD patients, miR-344b-1-3p was upregulated and controlled *TLR2*, *TNF*, and *IL1β* expression (83).

In order to analyze the effects of miRNAs on mechanism associated to these pathologies, structural lung cells, including ASMCs and airway epithelial cells, are implicated in the pathologic mechanisms of asthma, and their miRNA content have been studied. On the one hand, ASMCs play a critical role in asthma pathogenesis due to their abilities related to hypercontractility, proliferation, and secretion of inflammatory mediators. Dileepan and collaborators showed that miR-708 and miR-140-3p regulate the MAPK and PI3K signaling pathways associated to asthma immune response in human ASMCs (46, 51, 189). Later, the same group showed that miR-708 and miR-140-3p exert different effects in other proinflammatory genes, including *CCL2*, *CCL5*, *CCL11*, *CXCL8*, *CXCL10*, and *CXCL12* (47). Moreover, other miRNAs from ASMCs have been described, such as miR-145, miR-146a-5p, and miR-638, altering the functions of airway muscle cells (48–50).

On the other hand, airway epithelial cells are another cell type implicated in several processes of asthmatic pathogenesis, including airway remodeling, epithelial barrier repair, and production of several proinflammatory mediators (190). In this context, a number of miRNAs have been described in this cell type, regulating their functions or other pathological processes of asthma. In 2018, Zhang et al. investigated the role of miR-221 in airway epithelial cell injury in asthma (65). This miRNA was significantly increased in bronchial epithelial cells from asthmatic subjects compared to healthy controls and was implicated in epithelial cell injury in asthma by inhibiting *SIRT1* expression. However, there are several miRNAs that mitigate inflammatory status. Lambert and co-workers showed that miR-146a is released by airway epithelial cells in response to inflammatory stimuli like TNF-α (55). This fact constitutes an anti-inflammatory mechanism to enhance glucocorticoid effects. In addition, this miRNA, in conjunction with miR-146b, has been described as a negative regulator of inflammatory gene expression (*PTGS2* and *IL1B*) in lung epithelial and smooth muscle cells (56).

Other studies set out to find miRNAs that can be used to predict comorbidities; for instance, in blood, miR-210 expression can differentiate subjects with COPD and ischemic stroke from those with COPD or ischemia alone (191). MiR-1 reduction has been related to quadriceps skeletal muscle dystrophy in COPD (138). Plasma miRNAs can be used to identify patients with acute exacerbations of COPD such as miR-125b (105). PBMC-derived miRNAs are also differentially expressed in acute exacerbations of COPD compared to stable COPD and can differentiate between both conditions (129).

As previously described, plasma and exhaled breath condensate present differential miRNA profiles between asthma and COPD, which may be used to differentiate these diseases (100, 131). Nevertheless, having one of these respiratory diseases does not protect an individual against the other, so they may be present concomitantly. Asthma-COPD overlapping (ACO) is a condition where subjects present characteristics of both COPD and asthma, and it has been described in the Global Initiative for Chronic Obstructive Lung Disease-ACO guidelines (192). These subjects are normally defined as COPD subjects with eosinophilia (blood eosinophil count ≥200 eosinophils/µL) or asthmatics with chronic airway obstruction and smoking habit (≥20 pack per year) (193). Some miRNAs have been described as differentially expressed for ACO and can distinguish between asthma, COPD, and ACO. MiR-619-5p is downregulated in eosinophilic COPD subject serum compared to smoking and non-smoking asthmatics and COPD, and miR-4486 is differentially expressed in eosinophilic COPD when compared to non-smoking asthmatics, showing that even within the ACO group differences in miRNA expression can be found. The targets of this set of miRNAs include epidermal growth factors belonging to the ErbB signaling pathway associated to pathogenic inception of lung diseases and to the metabolism of xenobiotics by cytochrome P450 signaling pathways involved in ROS (194). Finally, our research group has showed that combined expression of miR-185-5p, miR-320a, and miR-21-5p was able to differentiate asthmatics from COPD and ACO with high sensitivity and specificity. Like in the previous article these deregulated miRNAs in asthma are able to regulated genes belonging to Erb2, MAPK, AMPK, and PI3K/AKT pathways that control cell proliferation and muscle contraction, alongside other targeted pathways as T-cell receptor, FoxO or TGF-β which are key in immune regulation. The regulation of those pathways by asthma specific miRNAs may account for the differences in asthma pathology compared to those other respiratory diseases (145).

In sputum, expression of miR-338 is higher in subjects with respiratory diseases (asthma, COPD, and ACO) compared to healthy subjects; similarly, miR-338 is higher in asthma than in COPD. The study by Lacedonia et al. also showed that miR-145 is increased in sputum supernatant of COPD and asthmatics *versus* controls, and that serum miR-338 levels are lower in ACO and COPD compared to healthy controls (70). Finally, miR-146a-5p, miR-10a-5p, and miR-31-5p have been shown to play a common role in both CRDs (94, 108).

Together, these works have demonstrated a variety of miRNAs dysregulated in asthma and COPD in relation to healthy subjects and promising results have been found, including the use of miRNAs as biomarkers. However, this is a broad field of research and many of the specific mechanisms and particular means of miRNA regulation in these respiratory diseases remain to be discovered, and systems biology can help to solve this enigma.

## MiRNAs and Treatments in Lung Diseases

### MiRNAs and Asthma Treatment

Glucocorticoids (GCs) remain the cornerstone of therapy for treating the inflammatory component of asthma and preventing asthma exacerbations. However, clinical response to GCs is complex and varies among individuals, as well as within the same individual, and some patients are resistant to this therapy. Different factors belonging to microenvironment can alter the canonical GC-induced signaling pathways, leading to reduced efficacy, collectively termed as sub-sensitivity, which include the entire spectrum of steroid-insensitivity and -resistance (195). Steroid sensitivity has been associated with different mechanisms, including dysregulated expression of GC receptor isoforms, neutrophilic inflammation and TH17 cytokines, oxidative stress-induced factors, and the downstream effect on histone deacetylase (HDAC) activation and gene expression. Recently, a new factor has been added in order to explain this phenomenon: the alterations in the expression of key transcription elements like miRNAs. Several studies conducted in this area, suggesting that circulating miRNAs may be useful potential biomarkers of asthma status or response to therapy (179).

In this sense, miR-155 has been the focus of different studies. Zhou et al. proposed that GCs may affect the inflammatory response by suppressing miR-155; these authors found that GCs attenuate lipopolysaccharide-induced inflammation and sepsis *via* downregulation of miR-155 expression (196), and forced miR-155 expression reverts the anti-inflammatory role of GCs (197, 198). In addition, miR-155-5p and miR-532-5p were identified as significantly associated with changes in dexamethasone-induced transrepression of NF-κβ. Authors identified these two functional circulating miRNAs predictive of asthma ICS treatment response over time, with an AUC of 0.86 (199).

Another miRNA that has been widely studied is miR-21. The study of Hammad et al. revealed a negative association between miR-21 and FEV_1_ post ICS treatment, which highlights the role in ICS treatment outcome as FEV_1_ reflects the grade of airway obstruction after ICS treatment (95). Elbehidy et al. found that miR-21 could be a novel predictor of ICS response, which helps in decision-making and identifying patients who are likely or unlikely to benefit from ICS therapy reducing the risk of side effects and sparing patients from the disappointment of treatment failure. MiR-21 had a predictive value in differentiating steroid-sensitive from steroid-resistant patients with an AUC value of 0.99 (200). Similar results were described by Wu et al. in 2014, who found that miR-21 expression was up-regulated in asthmatic adult bronchial epithelial cells regardless of treatment (201), but expression levels were decreased following ICS therapy (202).

Also, Kim et al. found that miR-21 drives severe, steroid-insensitive experimental asthma by amplifying PI3K-mediated suppression of HDAC2; thus, inhibition of increased miR-21 or PI3K responses suppresses disease and restores steroid-sensitivity (119).

Other studies investigating the expression profiles of 579 miRNAs in transgenic mice revealed that miRNAs were differentially expressed upon induction of experimental asthma following treatment with doxycycline and additionally suggested that miR-21 was the most up-regulated miRNA (66, 203).

Other authors reported an increase in infection-induced miR-9 in the airways of a mouse model and a similar increase in miR-9 in the sputum of neutrophilic asthmatics. These researchers therefore propose that miR-9 regulates glucocorticoid receptor signaling and steroid-resistance by reducing protein phosphatase 2A activity. Thus, blocking miR-9 function restores steroid sensitivity and suggests that this might serve as a novel approach for the treatment of steroid-resistant AHR (204).

One of the most important challenges may be to find biomarkers predicting treatment outcomes, and for this reason McGeachie et al. in 2017 investigated serum expression of 738 miRNAs in 160 children with asthma aged 5–12 years in search of predictors of asthma remission at the age of 14. The model, which was based on 12 variables including different miRNAs (miR-146b-5p, miR-106a, miR-126, and miR-30a), allowed prediction of remission with a sensitivity of 84% and a specificity of 70%. Thus, they hypothesize that miRNAs are potentially predictive biomarkers for treatment outcome (205).

However, not all authors agree on the role of miRNAs as regulators of GC treatment. Williams et al. sustain that changes in miRNA expression do not appear to be involved in the anti-inflammatory action of the corticosteroid budesonide (206). This discrepancy may be explained by the fact that the inflammatory changes were too mild, and by the degree of cellular heterogeneity in airway biopsies.

### MiRNAs and COPD Treatment

The main goal of pharmacologic COPD therapy is to treat the symptoms, reduce the frequency and severity of exacerbations, and improve tolerance and health status (207).

The main types of drugs normally used to treat COPD include long-acting β_2_-agonists, long-acting muscarinic antagonists, and ICS, which are the most widely used treatment as anti-inflammatory agents in COPD. However, a high percentage of COPD patients show a poor response to this therapy (208). It has now been recognized that current COPD treatments such as corticosteroids work, in part, through epigenetic mechanisms (209) and miRNAs is one of them (37).

In a recent manuscript authored by Faiz et al. (144) four miRNAs with changed expression after 6- and 30-month treatment with ICS compared with basal status (without any treatment) were identified. MiR-708 and miR-155 were downregulated and miR-320d and miR-339-3p were upregulated in both periods of time after treatment (6 and 30 months). Moreover, three were also altered in the same direction by ICS plus long-acting β2-agonists compared to placebo at 6 months of therapy: miR-320d, miR-339-3p, and miR-708; *in vitro*, these data were confirmed for miR-320d. Overexpression of miR-320d significantly reduced the IL-1β-induced activation of NF-κB signaling compared to miRNA negative control. Thus, the negatively correlated predicted targets of miR-320d are diminished by ICS treatment. So, this study identified four miRNAs affected by short- and long-term treatment with ICS compared to placebo in patients with moderate to severe COPD (144) and miRNAs associated with ICS therapy and inflammation provide relevant candidates as potential therapeutic targets in chronic inflammatory diseases.

In addition, the increase of HDAC2 could reduce GC insensitivity in some patients. Leuenberger et al. (110) showed that HDAC2 is directly targeted by miR-223 by binding to seed matches located in the 3’UTR of this mRNA transcript; in addition, the activity of total HDAC and HDAC2 in pulmonary endothelial cells is repressed in response to miR-223 overexpression. The reduced activity of this histone has been classically described in COPD patients and a significant inverse correlation between HDAC2 and miR-223 level has been observed in this COPD population (110). Therefore, this miR-223, through regulation of another epigenetic factor as the HDAC2, could interfere with treatment efficacy in COPD disease.

As commented previously, miR-146a has been described as an enhancer of the anti-inflammatory effects of GCs (55) and is negatively correlated with inflammation and Global Initiative for Chronic Obstructive Lung Disease stage in both stable and acute exacerbation COPD patients (210). COPD patients show an increased secretion of PGE_2_, which results in collagen overproduction and finally reduces lung capacity. In this sense, miR-146a expression is reduced in COPD patients and its target, COX-2, is simultaneously increased with a consequent increase of PGE_2_ levels (89). As COX-2 is sensitive to steroids and miR-146a target, this miRNA could contribute to the anti-inflammatory effect of this drug to reduce the increase of mucus and worsening of COPD evolution.

Moreover, as we have previously commented, COPD could have different etiologies such as tobacco or biomass smoke exposure. A recent manuscript from Velasco-Torres et al. on COPD due to biomass smoke exposure reported downregulation of miR-34a which implicates an activation of Notch 1 signaling. This finding is relevant because Notch 1 could represent an important target for therapy in these phenotypes of COPD (117).

Though the list is still small, several miRNAs have been modified by classical COPD treatments and, likewise, these miRNAs act over therapy targets and could contribute in different ways to treatment response in this respiratory pathology.

## Clinical Advances in the Use of MiRNAs

Finally, miRNA-based treatment has emerged as a potential approach for clinical intervention in some respiratory diseases such as asthma and COPD. It is based on miRNAs delivery in the specific site of action which constitutes one of the main aspects of development in relation to miRNA like therapeutic approach. A long list of miRNAs has been found to be linked to initiation, progression, or exacerbations in both respiratory diseases, especially in COPD. However, some have been studied more in depth, showing a high potential as future therapeutic tools through their up- or downregulation. In this sense, miR-146a (79, 89), miR-21 (113, 116), miR-150 (109), miR-145-5p (131, 148), miR-320d (132, 144), miR-155 (62) miR-223 (211), or miR-3162-3p (212) seem to hold promise as future elements in the therapeutic repertoire for COPD and asthma, respectively, some of which are common for both pathologies such as miR-146a (213–215) or miR-21 (54, 62).

All these examples show the never-ending list of miRNAs that, in the future, could potentially be a therapeutic approach in respiratory diseases. However, although the knowledge of miRNAs has grown exponentially in recent years, these data demonstrate that more studies are necessary before miRNAs can be employed as therapeutic tools. Currently, the idea of precision and personalized medicine is the objective for the near future, though this is a long and arduous path; thus, classical treatments continue to be the basis of therapy in asthma and COPD.

## Concluding Remarks

This review summarizes the previous knowledge about miRNAs in chronic respiratory diseases as asthma and COPD, trying to be the first step forward for the compilation and application of systems biology approaches for understanding their roles. Many miRNAs present differential expression in diverse samples, and are known for their capability of being biomarkers or for having a specific role in the pathogenesis of these diseases, but the current approaches are unsuited for giving a systemic level view for data interpretation.

For overcoming this issue, systems biology may be the optimal tool. By the combination of data-driven model elaboration and model-driven experimental design, researchers might be able to elucidate how miRNAs work together in disease pathogenesis and diagnosis, giving the full picture view of miRNA regulatory system.

## Author Contributions

JC, JR-M, BS, MG-M, NR, and VP conceived of the review and wrote the manuscript. JC, JR-M, and VP prepared the figures. The review was performed under the supervision of JC and VP. All authors contributed to the article and approved the submitted version.

## Funding

This manuscript was supported by ISCIII – Instituto de Salud Carlos III, Fondo de Investigacioìn Sanitaria – FIS and FEDER (Fondo Europeo de Desarrollo Regional) [PI15/00803, PI18/00044 and FI16/00036], CIBERES, Merck Health Foundation funds and RTC-2017-6501-1 (Ministerio de Ciencia, Innovacioìn y Universidades).

## Conflict of Interest

VP has received honoraria (advisory board, speaker) and/or institutional grant/research support from Astra-Zeneca and GSK.

The remaining authors declare that the research was conducted in the absence of any commercial or financial relationships that could be construed as a potential conflict of interest.

## Abbreviations

α-SMA, alpha-smooth muscle actin; ACO, Asthma-COPD overlapping; ASMC, airway smooth muscle cells; AUC, area under curve; BALF, bronchoalveolar lavage fluid; COPD, chronic obstructive pulmonary disease; COX-2. cyclooxygenase-2; CRDs, chronic respiratory diseases; CSE, cigarette smoke extract; FEV1, forced respiratory volume in the first second; GCs, glucocorticoids; HBECs, human bronchial epithelial cells; HDAC, histone deacetylase; HIF-1α, hypoxia-inducible factor 1-alpha; ICS, inhaled corticosteroid; IFN-γ, interferon gamma; IL, interleukin; lncRNA, long-noncoding RNA; MAPK, mitogen-activated protein kinase; miRNAs, microRNAs; PBMCs, peripheral blood mononuclear cells; PGE2, prostaglandin E2; PI3K, phosphatidylinositol 3-kinase; ROS, reactive oxygen species; TGF, transforming growth factor; TLR, Toll-like receptor; TNF, tumor necrosis factor.
