# Corrigendum: MicroRNAs as potential regulators of immune response networks in asthma and chronic obstructive pulmonary disease

**DOI:** 10.3389/fimmu.2022.1051260

**Published:** 2022-10-13

**Authors:** José A. Cañas, José M. Rodrigo-Muñoz, Beatriz Sastre, Marta Gil-Martinez, Natalia Redondo, Victoria del Pozo

**Affiliations:** ^1^ Immunoallergy Laboratory, Immunology Department, Instituto de Investigación Sanitaria Fundación Jiménez Díaz (IIS-FJD), Madrid, Spain; ^2^ CIBER de Enfermedades Respiratorias (CIBERES), Madrid, Spain

**Keywords:** chronic respiratory diseases, systems biology, microRNAs, asthma, chronic pulmonary obstructive disease

In the published article, there was an error in the **Funding** statement. The **Funding** statement for the article was depicted as: “This manuscript was supported by Fondo de Investigacioín Sanitaria – FIS and FEDER (Fondo Europeo de Desarrollo Regional) [PI15/00803, PI18/00044 and FI16/00036], CIBERES, Merck Health Foundation funds and RTC-2017-6501-1 (Ministerio de Ciencia, Innovacioín y Universidades).” The correct Funding statement appears below.

## Funding

This manuscript was supported by ISCIII – Instituto de Salud Carlos III, Fondo de Investigacioìn Sanitaria – FIS and FEDER (Fondo Europeo de Desarrollo Regional) [PI15/00803, PI18/00044 and FI16/00036], CIBERES, Merck Health Foundation funds and RTC-2017-6501-1 (Ministerio de Ciencia, Innovacioìn y Universidades).

The authors apologize for this error and state that this does not change the scientific conclusions of the article in any way. The original article has been updated.

## Publisher’s note

All claims expressed in this article are solely those of the authors and do not necessarily represent those of their affiliated organizations, or those of the publisher, the editors and the reviewers. Any product that may be evaluated in this article, or claim that may be made by its manufacturer, is not guaranteed or endorsed by the publisher.

